# Potentiating Cancer Immune Therapy *via* Nanomaterials and Purinergic Signaling

**DOI:** 10.3389/fcell.2022.893709

**Published:** 2022-05-04

**Authors:** Davide Ferrari, Stefania Gessi, Stefania Merighi, Manuela Nigro, Alessia Travagli, Jorge S. Burns

**Affiliations:** ^1^ Section of Microbiology and Applied Pathology, Department of Life Science and Biotechnology, University of Ferrara, Ferrara, Italy; ^2^ Department of Translational Medicine and for Romagna, University of Ferrara, Ferrara, Italy; ^3^ Department of Environmental and Prevention Sciences, University of Ferrara, Ferrara, Italy

**Keywords:** cancer immune therapy, nanomaterials, tumor microenvironment (TME), A_2A_ adenosine receptor, P2X7 (purino) receptor

## Introduction

Adenosine, an autacoid nucleoside interacting with P1 receptors, activates four G protein-coupled receptors named A1, A_2A_, A_2B_, and A_3_, crucially regulating several human pathologies (Borea et al., 2018). It affects both neoplastic and immune cells, promoting cancer cell proliferation, neo-angiogenesis, immunoescape, and metastasis (Arab and Hadjati, 2019). Extracellular nucleotides such as ATP, ADP, and UTP also function as cell-to-cell communication signals by binding and activating P2 receptors belonging to the P2X and P2Y subfamilies (Kennedy, 2021). These receptors are further subdivided into different subtypes (Khakh et al., 2021). The differential expressions of P1 and P2 receptors both in immune and tumor cells generate a complex picture. Cancers are able to convert extracellular ATP into immunosuppressive adenosine, through the activation of CD39 ectonucleotidase that hydrolyzes ATP to AMP, and a subsequent CD73 enzyme that transforms AMP into adenosine, with the stimulation of adenosine receptors on immune cells activating numerous immunosuppressive effects (Borea et al., 2018; Boison and Yegutkin 2019). The shift from P2 to P1 activation is important for limiting the inflammatory response, thus preventing tissue damage, but may also deleteriously inhibit immunosurveillance (Antonioli et al., 2013; Allard et al., 2019). Targeting CD39 and CD73 has, therefore, become a new way to fight cancer (Perrot et al., 2019; Moesta et al., 2020; Li et al., 2019). This review conjugates the current knowledge of purinergic signaling in cancer biology with techniques involving nanomaterials to increase anticancer immune responses.

### P1 Receptors and Cancer

Two hallmarks connecting adenosine to cancer include 1) solid tumors develop hypoxia and increase adenosine from nanomolar to micromolar concentrations and 2) the A_2A_ receptor is an essential brake of immune cells (Sitkovsky M. V., 2020; Hatfild and Sitkovsky, 2020). The hypoxic activation of the master oxygen-sensitive transcriptional regulator HIF-1α upregulates ecto-5′-nucleotidase (CD73), generating adenosine accumulation associated with poor prognosis in many neoplasms (Borea et al., 2017). Adenosine activates cAMP-elevating A_2A_ receptors to inhibit CD8^+^, CD4^+^ lymphocytes, and natural killer (NK) cells but stimulates B and T regulatory lymphocytes (Treg), tumor-associated macrophages (TAMs), and myeloid-derived suppressor cells (MDSCs), thus establishing a typically immunosuppressive tumor microenvironment (TME) (Vijayan et al., 2017). This encouraged immunologists to recognize adenosine as a new “immune checkpoint regulator” that stimulated the classic anti-cytotoxic T-like antigen 4 (CTLA4) and anti–programmed death-ligand 1 (PD-L1) to increase immunoescape (Sitkovsky M. V., 2020). Indeed, CTLA4 and PD-L1 inhibitors have been well-tolerated in cancer patients, improving overall morbidity and survival versus standard chemotherapy. However, efficacy may be limited to relatively few patients in some tumor types, reflecting the presence of alternative immunosuppressive factors in TME. Notably, anti-PD1 therapy increased immunosuppressant A_2A_ receptors on CD8^+^ T cells; moreover, patients resistant to immunotherapy showed CD73 upregulation, suggesting that adenosine machinery counteracted the effects of immune checkpoint inhibitor drugs (Zarek et al., 2008). One improvement strategy has been implemented to inhibit (Kotulová et al., 2021) the hypoxia-HIF-1α-A_2A_ receptor-mediated pathway in the TME through A_2A_ receptor antagonists (Hatfield and Sitkovsky, 2020; Willingham et al., 2020). Accordingly, genetic silencing of the A_2A_ receptor strongly increased inflammation and tumor rejection in mice (Ohta and Sitkovsky, 2001; Ohta et al., 2006; Sitkovsky M. V., 2020). A series of phase I/II clinical trials, evaluating the safety and efficacy of A_2A_ receptor blockers/CD73 inhibitors including oleclumab, CPI-006, BMS-986179, and NZV-930 and A_2A_ receptor antagonists such as ciforadenant, inupadenant, taminadenant, AZD4635, and preladenant alone or coadministered with immune checkpoint inhibitors such as anti-PD1 or anti-PDL1, are under evaluation (Arab and Hadjati, 2019; Arab et al., 2021; Franco et al., 2021; Thompson and Powell, 2021).

Beyond targeting the A_2A_ receptor, anticancer immunotherapy can also be potentiated by inhibiting the A2B receptor, a subtype also capable of stimulating cAMP in T cells. Phase I clinical trials of A2B blockers in patients with advanced cancer are underway (Franco et al., 2021). Arguably, this pharmacological approach might only succeed in patients bearing hypoxic tumors with a sufficient number of tumor-reactive T cells, yet this consideration remains to be resolved (Sitkovsky M. V., 2020; Fong et al., 2020).

### P2 Receptors and Cancer

The TME is rich in ATP and its metabolites modulating tumor and immune cell biology and responses (Di Virgilio et al., 2018). The contribution of P2 receptors to cancer biology has been intensively investigated (Chiarella et al., 2021). The ATP-activated P2X7 receptor has emerged as a pivotal membrane molecule in tumors as it is expressed by cancer cells and by macrophages, dendritic cells, and lymphocytes infiltrating the tumor mass (De Marchi et al., 2019).

Tumor cell cytotoxicity (apoptosis or necrosis) due to prolonged P2X7 receptor activation and pore formation was a desirable anti-tumor response of this membrane molecule (Feng et al., 2006; Fu et al., 2009; Bian et al., 2013; Avanzato et al., 2016). However, subsequent identification of P2X7 receptor variants, with more precise characterization of the responses and measurement of cancer cell expression levels, indicated this subtype was upregulated in many tumor types (McLarnon, 2017; Di Virgilio et al., 2018, Zhang et al., 2019a; 2019b). More significantly, P2X7 receptor stimulation by low extracellular ATP concentrations was pro-tumorigenic, favoring cancer cell survival, proliferation, motility, and chemoresistance (Adinolfi et al., 2012; Schneider et al., 2015; Arnaud-Sampaio et al., 2020). In addition to the P2X7 receptor subtype, the P2X4, P2X5, P2Y_6_, and P2Y_12_ receptors also have involvement in tumor biology (Roger et al., 2015). P2X4 and P2X7 receptor subtype expressions concurred with tumor cell proliferation (He et al., 2020). In contrast, P2X5 receptor mediated an anti-proliferative (Zhang et al., 2020) effect by inducing tumor cell differentiation. Cumulative reports have indicated pro-neoplastic P2Y_2_ receptor-mediated responses conferring resistance to cell apoptosis, stimulation of tumor replication, and dissemination (Limami et al., 2012; Choi et al., 2013; Schumacher et al., 2013). The lack of expression of the P2X7 receptor in P2X7 KO mice induced a decrease in CD8^+^ lymphocytes while the number of Treg cells increased (De Marchi et al., 2019).

**FIGURE 1:**
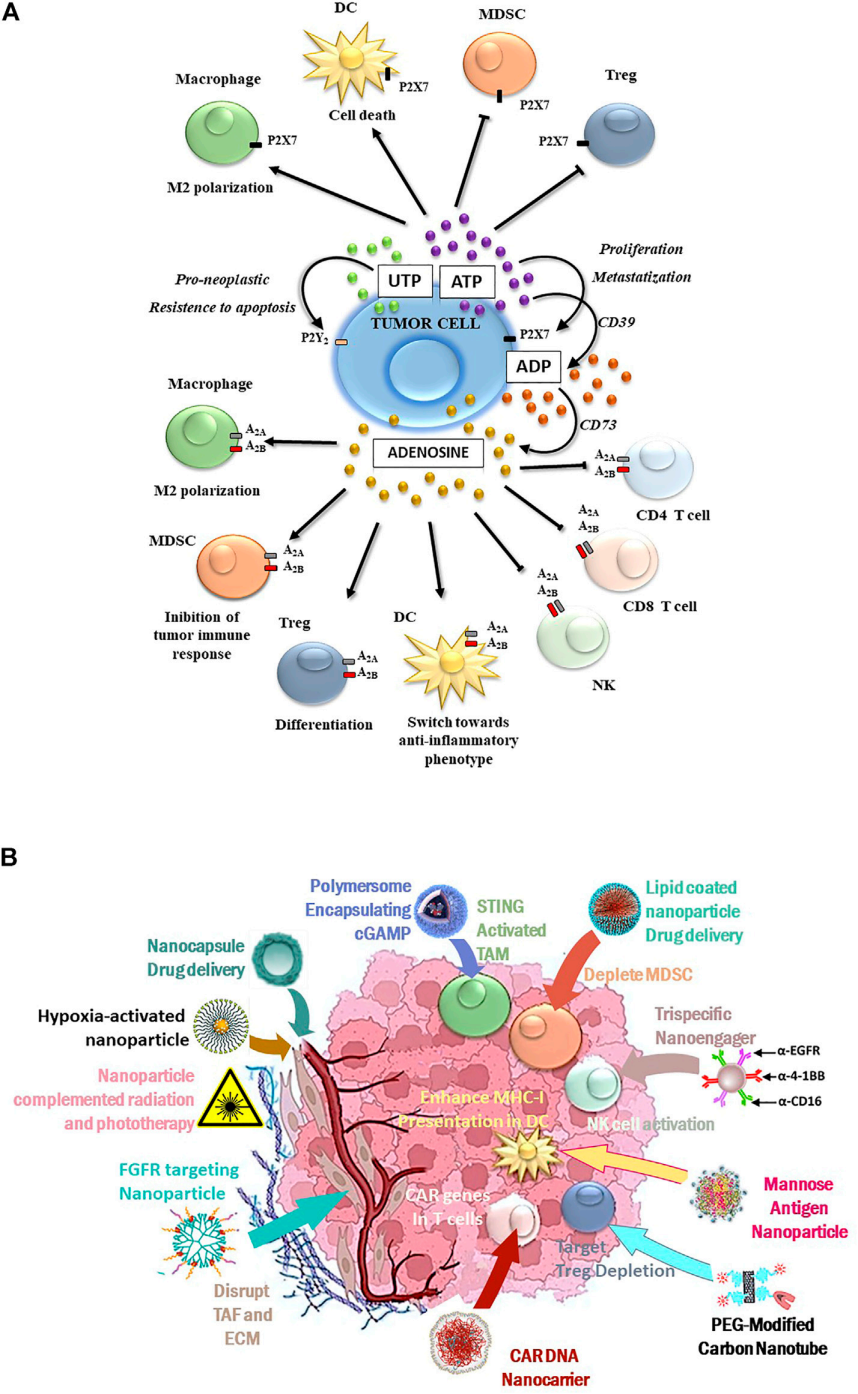
**(A)** Purinergic-mediated responses occurring in the TME: P2 receptor-induced activities are summarized in the upper part of the figure, while P1 receptor-mediated responses are depicted in the lower figure part. **(B)** Schematic diagram for tailored nanoparticles targeting the TME and its immunological components to potentiate cancer immune therapy.

From a pharmacological and therapeutic perspective, P2 receptors have high potential to complement radiation therapy against resistant, highly malignant cancers. The stimulation of P2X7, P2Y_6_, and P2Y_12_ receptors was significant in the DNA damage response induced by *γ*-irradiation of adenocarcinoma A549 cells (Ide et al., 2014). B16 melanoma cells both *in vitro* and *in vivo* responded similarly to P2X7 receptor antagonists (Tanamachi et al., 2017). The use of single P2 receptor subtype inhibitors was often sufficient to block tumor cell growth and dissemination (Drill et al., 2021). The growth of human high-grade gliomas was inhibited by P2X7 subtype antagonists (Kan et al., 2020); receptor inhibitors, such as emodin, and the *Uncaria tomentosa* extract effectively counteracted the P2X7 receptor-mediated breast cancer spread (Zhu et al., 2021). P2X7 receptor antagonization could also usefully reduce pain in cancer patients with metastases. In particular, the P2X7 receptor antagonists AFC5261 and A-740003 were promising in animal models (Li et al., 2018; De Marchi et al., 2019; Falk et al., 2019). Further identification and characterization of new P2X7 receptor modulators and inhibitors were recommended (Hempel et al., 2013). Also for consideration, the expression of P2X7 and other P2 receptors by immune cells participated in immunosurveillance (Jelassi et al., 2013; Grassi and Conti, 2021). The awareness of the importance of P2-mediated signaling in cancer pathogenesis and progression (Figure 1A) has prompted therapeutic strategies targeting extracellular nucleotides. In this light, nanomaterials may improve anticancer outcomes by modulating the immune and tumor cell purinome.

### Nanomaterials and Immunosurveillance

Although TME immunosurveillance may be markedly heterogeneous, most anticancer agents rely on the reactivation of homeostatic immune defense mechanisms (Joyce and Fearon, 2015; Terry et al., 2017; Ni et al., 2021). Initially, innovative nanomaterials improved upon conventional treatments yet soon drew criticism when nanoparticles elicited toxic effects from immunological alterations (Lenders et al., 2020). Nonetheless, rationally tailored nanomaterials have renewed interest in penetrant TME modulators (Zhang et al., 2021) that address tumor immune evasion (Guevara et al., 2021) by immunotherapy enhancement to promote immunogenic tumor cell death (Aikins et al., 2020) (Nogrady, 2021). The multiple cell types comprising the TME provide alternative nanomaterial targets, and their involvement in intervention design can be reciprocal (Song et al., 2017). For example, to counteract tumor adenosine accumulation, lipid nanoparticles mediating the knockdown of the corresponding A_2A_ receptor in memory T cells could rescue CD8^+^ T-cell chemotaxis for infiltration into the TME of head and neck squamous cell carcinomas (Newton et al., 2021). Nanoparticle-based delivery approaches also include cell membrane–camouflaged nanocarriers (Grimaudo, 2021) such as tumor-associated macrophage membrane-coated nanoparticles (Chen et al., 2021). Cell membrane-bioinspired nanoparticles can provide superior immune regulation, nanocapsule drug delivery (Zhang et al., 2019c; Irvine and Dane, 2020), tumor targeting, and biocompatibility (Mu et al., 2021).

Yet diversity among tumorigenic cells and between individuals may still Yet, thwart nano-based delivery systems. The improved knowledge of various chronological stages of TME development remains necessary for more effective nanoplatform implementation (Yang et al., 2021) to target the more persistent subpopulation of cancer stem cells (Duan et al., 2021). The highest immunotherapeutic efficacy occurs when nanoparticles achieve precise and timely delivery, specifically targeting neoplastic cells with minimal harm to healthy cells (Muluh et al., 2021). Addressing TME traits, hypoxia-activated nanoparticles have theranostic applications (Wang et al., 2019). Since TME hypoxia blocked antitumor immunity (Singleton et al., 2021), tumor hypoxia-activated polymeric micelles were used to both activate strong cytotoxicity and stimulate a systemic antitumor immunity that effectively eradicated breast cancer in preclinical murine models (Liu et al., 2021). Hypoxia-modifier nanoparticles (Yuan et al., 2021) targeting the blood–brain barrier, enhanced immunotherapy of glioblastoma (Meng et al., 2021), a particularly aggressive form of cancer involving intracellular purine alterations (Debom et al., 2021; Giuliani et al., 2021). Cancer metastasis treatment remains a highlight of nanomedicine-based immunotherapy (Zhang et al., 2019). Excellent efficacy was observed for TME-activated nanoparticle chemodynamic immunotherapy of melanoma-derived lung metastasis (Zhai et al., 2021).

### How Nanomaterials Can Be Used to Modulate TME Purinergic Signaling

Compared to the relatively heterogenous tumor-cell population, non-tumorigenic supportive cells within the TME such as tumor-associated fibroblasts (TAFs) may present a more consistent target for nanoparticle intervention (Li et al., 2021), yet some limitations persist. nanomaterial-based TME modulation impinging upon purinergic signaling pathways can serve to additionally recruit the immune system to provide more integrative therapy (Laplane et al., 2019; Shi and Lammers, 2019). Nanomaterials can be adapted to modulate purinergic signaling in a number of ways since nanoparticles can be size-tailored to have diameters that match pore sizes present in leaky TME vasculature, thus establishing size-related penetration and accumulation (Yu et al., 2020). Moreover, nanoparticles can assist with improved delivery of drugs such as A_2A_ antagonists that counteracted immunosuppression (Arruga et al., 2021). It is notable that the purinergic signaling network is subjected to modulation by microRNA (miRNA) (Ferrari et al., 2016), and over 30 miRNAs directly or indirectly modulate P1 and P2 receptors and ectoenzymes, with miR-187 capable of modulating both P2X7 and CD73 (Guo et al., 2022). Notably, miRNA that bind the 3′ untranslated region of the P2X7 receptor can affect the development of breast cancer by influencing the P2X7 receptor expression (Zhu et al., 2021). Nanoparticles are well-suited for precision medicine strategies to deliver purinergic signaling-specific miRNA and silencing RNA (siRNA) therapeutics (Kara et al., 2022). It has already been demonstrated that the nanoparticle delivery of siRNA-CD73 to the central nervous system blocked the CD73 expression in the glioblastoma immune microenvironment, inducing apoptosis to delay tumor growth (Azambuja et al., 2020). Smart nanomaterials can be engineered to exploit TME-specific purinergic pathway anomalies. A hydrogel of alginate conjugated with an ATP-specific aptamer hybridized with immunoadjuvant CpG oligonucleotides enabled the release of immune adjuvants in synchrony with low-dose repeated chemo/radiotherapies. This achieved a remarkable synergistic response; in addition to eliminating tumors, the evoked immune memory rejected re-challenged tumors and inhibited distant tumor metastases when combined with immune checkpoint blockade (Sun et al., 2021).

### Nanoparticles Modulating TME Purinergic Pathways Potentiate Immune Therapy

Innate immune interactions include macrophage responsiveness to damage-associated molecular patterns (DAMPs) originating from the cancer cells. M2-like tumor-associated macrophages (TAMs) can efficiently engulf neighboring apoptotic cells abundant in solid tumors, an early immunosuppressive mechanism preventing a DAMP-mediated immune response. The MER proto-oncogene tyrosine kinase (MerTK) can promote an “eat me” signal on dying cells to enhance efferocytosis (Ou et al., 2021). Consequently, apoptotic cells are eliminated before releasing intracellular ATP and cyclic GMP that would otherwise activate the ATP-gated P2X7 channels of TAMs and also cytosolic nucleic acid sensor pathways, including cyclic GMP-AMP synthase (cGAS) producing cyclic guanosine monophosphate–adenosine monophosphate (cGAMP), a second messenger binding and activating the adapter protein, stimulating interferon gene (STING), expressed in TAMs and other cells of the TME. The production of stress-responsive cytokines would ultimately cause M2 macrophages to be polarized toward an immune-activated M1 phenotype (Zhao et al., 2021). Appropriately, macrophages have become key targets for nanoparticle intervention (Medrano-Bosch et al., 2021). A nanoparticle-incorporating STING activator cGAMP enhanced the antitumor immunity in PD-L1-insensitive models of triple-negative breast cancer (Cheng et al., 2018) and improved the clinical outcome of immunotherapy for melanoma (Shae et al., 2019). Cationic silica nanoparticles induced necrotic cell death and activation of the STING in the TME to enhance antitumor immunity (An et al., 2018). Inhalable nanoparticulate agonists of STING synergized with radiotherapy to provide the long-term control of lung metastases (Liu et al., 2019). Combining nanoparticles with compatible forms of therapy such as radiation therapy (Huang et al., 2021) or photodynamic therapy (Jin et al., 2021) improved antitumor efficacy by promoting immunogenic cell death.

Nanomaterials are also capable of enhancing the trained acquired immune response (Magadán et al., 2021), and they have been rationally designed to enhance T-cell expansion, navigate physical barriers, and modulate the TME to overcome barriers to T-cell-based immunotherapies (Gong et al., 2021). Engineered immunomodulating nano-adapter particle rafts such as trispecific natural killer cell nanoengagers (Au et al., 2020) carry more than one monoclonal antibody (mAb) to bridge effector and tumor cells. More effective responses than simply mixing the parental mAbs with T cells, NK cells, natural killer (NK) cells, or macrophages were observed (Jiang et al., 2021). Nanogels selectively released an interleukin-15 cargo upon T-cell receptor activation and expanded T cells in tumors 16-fold relative to the systemic administration of free cytokines. The higher doses of cytokines could be administered, without toxic side effects, to potentiate human chimeric antigen receptor (CAR)-T cell therapy (Tang et al., 2018). Nanoparticle versatility, exemplified in Table 1 and Figure 1B, has meant that numerous clinical nanomaterials and drugs potentiating immunotherapy are currently under development (Li et al., 2020; Hu and Huang, 2022).

**TABLE 1:** Examples of immunomodulatory nanoparticle types, tumor microenvironment (TME) interactions and co-involved purinergic pathways.

Nanomaterial	Size (nm)	TME target	Co-involved purinergic ecto-enzyme receptor subtype	Reference
CAR DNA Nanocarrier	155 ± 40	murine CD8^+^ T cell	P2X7	Smith et al. (2017)
FGFR targeting nanoparticle	10-200	Tumor Associated Fibrobasts (TAF)	CD73	Li et al. (2021)
Hypoxia-activated nanoparticle	254 ± 27	Hypoxia	CD39, CD73, A_2A_, P2Y_2_, P2X7, P2Y_11_	Wang et al. (2019)
Lipid coated nanoparticle drug delivery	≈30	Myeloid-Derived Supressor Cells (MDSC)	P2X7, A_2B_	Zhang et al. (2019)
Mannose antigen nanoparticle	210	Dendritic Cell (DC	P2X7	Pei et al. (2021)
Nanocapsule drug delivery	100-200	Tumor Extracellular Matrix	P2X4, P2X7, P2Y_12_	Irvine and Dane, (2020)
PEG-modified carbon nanotube	101 ± 41	Regulatory T cells (T_reg_)	A_2A_, A_2B_	Sacchetti et al. (2013)
Polymersome encapsulating cGAMP	20-100	Tumor Associated Macrophage (TAM)	P2X7	Shae et al. (2019)
Trispecific nanoengager	112 ± 7	Natural Killer (NK) cell	CD39, A_2A_, A_2B_, A_3_, P2X7	Au et al. (2020)

## Discussion

The TME, heavily conditioned by nucleotide/nucleoside release and hydrolysis, makes purinergic signaling an extremely attractive target for strategic modulation of both cancer and immune cells, but responses to antagonists or agonists are highly context-dependent (Hreich et al., 2021). The inhibitors of specific purinome components have successfully blocked tumor progression and metastasis in animal models and preclinical studies, yet improved specific therapeutic strategies are needed. The recent implementation of nanomaterials has shown that they can be very effective agents, acting on their own, delivering mRNA or improving mAb presentation to disrupt the TME refractoriness to immune therapy.
